# Occurrence of Virulence Genes Associated with Diarrheagenic *Escherichia coli* Isolated from Raw Cow’s Milk from Two Commercial Dairy Farms in the Eastern Cape Province, South Africa

**DOI:** 10.3390/ijerph111111950

**Published:** 2014-11-18

**Authors:** Lesley-Anne Caine, Uchechukwu U. Nwodo, Anthony I. Okoh, Roland N. Ndip, Ezekiel Green

**Affiliations:** 1 Departments of Biochemistry and Microbiology, University of Fort Hare, Private Bag X1314, Alice 5700, South Africa; E-Mails: 200906584@ufh.ac.za (L.C.); UNwodo@ufh.ac.za (U.U.N.); AOkoh@ufh.ac.za (A.I.O.); RNdip@ufh.ac.za (R.N.N.); 2 Department of Microbiology and Parasitology, Faculty of Science, University of Buea, P.O. Box 63, Buea, Cameroon; E-Mail: ndip3@yahoo.com

**Keywords:** *Escherichia coli*, virulence markers, raw milk

## Abstract

*Escherichia coli* remains a public health concern worldwide as an organism that causes diarrhea and its reservoir in raw milk may play an important role in the survival and transport of pathogenic strains. Diarrheagenic *E. coli* strains are diverse food-borne pathogens and causes diarrhea with varying virulence in humans. We investigated the prevalence of pathogenic *E. coli* in raw milk from two commercial dairy farms. Four hundred raw milk samples, 200 from each dairy farm, were screened for the presence of *fliCH7, eagR, ial, eagg, lt,* and *papC* genes. In dairy farm A, 100 E. coli were identified based on culture, oxidase and Gram staining, while 88 isolates from dairy farm B were identified in the same manner. Gene detection showed *fliCH7* 27 (54%) to be the highest gene detected from farm A and *lt* 2 (4%) to be the lowest. The highest gene detected in dairy farm B was *fliCH7* 16 (43.2%) and *papC* 1 (2.7%) was the least. The amplification of pathogenic genes associated with diarrheagenic *E. coli* from cows’ raw milk demonstrates that potentially virulent *E. coli* strains are widely distributed in raw milk and may be a cause of concern for human health.

## 1. Introduction

*Escherichia coli* is a commensal in the intestines of a variety of animals, including man. However, diarrhoeal diseases caused by the pathogenic strains of this species can be debilitating and sometimes fatal [1]. The mechanisms involved in the disease processes and virulence attributes have been used to differentiate pathogenic strains of *E*. *coli* and is divided into diarrhoeal pathogens causing diarrhoea (diarrhoeagenic *E. coli*) and extraintestinal *E. coli* (ExPEC) [2] based on the site of infection. However, *E. coli* pathotypes may cause three main types of infections: sepsis/meningitis, enteric and diarrheal diseases, and urinary tract infections [3]. 

Enteric pathogens have been broadly divided into enterotoxigenic *E. coli* (ETEC), enteropathogenic *E. coli* (EPEC), enteroinvasive *E. coli* (EIEC), enterohaemorrhagic *E. coli* (EHEC) have been established, while enteroaggregative *E. coli* (EAEC), diffusely adherent *E. coli*, necrotoxic *E. coli*, and cell-detaching *E. coli* [4]. Two additional *E. coli* pathotypes, collectively called extraintestinal pathogenic *E. coli* [5] are responsible for extraintestinal infections. Extraintestinal pathogenic *E. coli* consist of uropathogenic *E. coli* (UPEC) causing urinary tract infections, *E. coli* strains that causes septicaemia, and neonatal meningitis associated *E. coli* (MNEC) [6]. In all *E. coli isolates* the *uid*A gene, which codes for the β-glucuronidase enzyme, serve as a target for fluorogenic PCR detection of *E. coli* in approximately 95% of naturally occurring *E. coli*.

Epidemiology, treatment, pathogenesis, and clinical manifestations can be used to preliminary identify each pathotype [7]. However, more researchers are using molecular methods such as polymerase chain reaction to identify pathotypes. EPEC pathotype has been sub-divided into typical and atypical EPEC depending on their adhesion mechanisms in human epithelial cells [8]. Typical EPEC’s adhesion is mediated by *bfpA* found in the EPEC adhesion factors type plasmid (EAF) that makes the organism to adhere to human epithelia cells in a localized manner [8]. The presence of locus of enterocyte effacement has been used to identify atypical EPEC. This locus codes for the gene of attaching and effacing, *eae*. This gene enables the bacteria to adhere and destroy human intestinal enterocytes [9].

Enteroaggregative *E. coli*, producing enteroaggregative mechanisms that promotes diarrhea in humans are coded for by the e*ag*R, the master regulator of EAEC virulence, controls the expression of adherence factors, a dispersin protein, and a cluster of genes encoded on the EAEC chromosome. One of the most common clinical manifestations of EAEC infection is watery diarrhea, usually unaccompanied by blood or mucus.

EIEC is characterized by the destruction of the colonic epithelium caused by the inflammatory response induced upon invasion of the mucosa by EIEC [10] using the *ial* (marker for invasivity) gene. ETEC are important causes of total diarrheal episodes worldwide. These strains cause diarrhea through the action of enterotoxins: the heat-labile (LT) and heat-stable (ST) enterotoxins. Some strains may express an LT only, an ST only, or both an LT and an ST coded for by *lt and st* in the plasmid. Adenylate cyclase is activated by LT, causing diarrhea while guanylyl cyclase in intestinal epithelial cells is activated by ST causing fluid secretion. *E. coli* strains that are capable of producing both LT and ST toxins (ETEC) can cause severe diarrhea [11]. 

Enterohaemorrhagic *E. coli* (EHEC) is associated with foodborne outbreaks in the developed world and can cause bloody diarrhoea, haemorrhagic colitis (HC) and the Haemolytic Uraemic Syndrome (HUS) due to the elaboration of Shiga toxin (Stx). In EHEC strains, stx-genes are typically found in transmissible lambdoid bacteriophages [12]. Other virulence factors such as membrane protein intimin, encoded by the *eae* gene, *fliC*H7 encoding flagella antigen H7 and enterohaemolysin, encoded by the *HlyA* gene [13] have been identified. 

Virulence factors such as toxins (cytotoxic necrotizing factors and hemolysin), adhesins (P fimbriae, afrimbriae, type 1 fimbriae, S and F1C fimbriae), polysaccharide coatings (group II capsules) and siderophores (the aerobactin system) [14,15], associated UPEC are thought to be responsible for the pathogenic potential of the organism. Specific adhesins that mediates specific adhesion can be differentiated based on the receptor binding specificity. The most significant mannose-resistant adhesins are the P fimbriae encoded by *E. coli* pap (pyelonephritis associated pilus) operon. However, they are expressed by few *E. coli* serotypes [16]. Uropathogenic *E. coli* strains have been identified by detecting urovirulence factors including those mentioned earlier using conventional PCR [15].

Discriminating various *E. coli* pathotypes from cattle and their products have been greatly studied in the last two decades with biomolecular techniques favoured over serology in detection of *E. coli* pathotypes because they are more sensitive and specific [17,18,19,20,21]. It therefore became the objective of this study to find the prevalence of *E. coli* virulence genes from two dairy farms in the Eastern Cape, South Africa. 

## 2. Experimental Section

### 2.1. Study Sites

Eastern Cape Province is one of the poorest and second largest provinces in South Africa and mainly comprised of rural settlements, it is also the “livestock” province of the country carrying 46% of South Africa’s goats, 28% sheep, and 21% cattle. Dairy farm A has been in operation since 2007, it has 800 dairy cows and produces about 10,000 L of milk per day which is bought by one of the biggest milk companies in South Africa. Dairy farm A has a rotary milking parlour whereas dairy farm B has an in-line milking system. Both dairy farms are located in the Eastern Cape Province. Dairy farm B has been in operation since 2008, it has 600 dairy cows and produces about 8000 L of milk per day. 

### 2.2. Isolation and Identification

We collected 400 raw milk samples from individual lactating cows showing clinical and subclinical signs of mastitis from the chosen two dairy farms (200 (100 from clinical and 100 from subclinical mastitis) from farm A and 200 (100 from clinical and 100 from subclinical mastitis) from farm B). Clinical mastitis occurs when visible signs of inflammation are observed in the udder of the cow or the teats; while Subclinical mastitis is more subtle in that no signs of infection are visible this requires regular monitoring and laboratory testing. Subclinical mastitis was determined using California Mastitis Test. Samples were collected according to the in-house procedure on each farm. Briefly, each milking station has a small collection bottle with a little hole for sample collection that is open and closed using a tap. The bottles are washed after each individual cow’s milking session. The samples were collected from that tap. The samples were cultured on selective medium Violet red bile—mug agar (Merck, Johannesburg, South Africa) and incubated at 37 °C for 24 h. The plates were checked under UV light at 360–370 nm after 24 h of incubation. Light blue fluorescence indicates the presence of *E. coli* [22,23]. Biochemical tests including Gram staining, catalase and oxidase test were performed. Those organisms that were Gram negative, catalase positive and oxidase negative were identified using API 20E (BioMerieux, Marcy I’Etiole, France) following manufacturer’s instructions. 

### 2.3. DNA Extraction

DNA was extracted from identified *E. coli* and from a positive control strains for *E. coli* (ATCC 8739, South African Bureau of Standards (SABS), No. ESC 20) purchased from South African Bureau of Standards, Pretoria, South Africa. DNA extraction was done following the method of Maugeri *et al.* [24] and Lopez Saucedo *et al.* [25]. Briefly, single colonies of presumptive *E. coli* grown overnight at 37 °C on EMBA plates were picked, suspended in 200 μL of sterile distilled water, vortexed for 2 min using MS2 Minishaker (Digisystem Laboratory Instruments Inc., New Taipei City, Taiwan). The cells were then centrifuged for 10 min at 13,000 rpm (ThermoFisher Scientific, Schwerte, Germany). Five hundred microliters (500 µL) distilled water was pipetted into the eppendorf tubes, vortexed and the cells were lysed using a heat on Dri-Block DB.2A (Techne, Johannesburg, South Africa) for 15 min at 100 °C. The cell debris were removed by centrifugation at 10,000 rpm for 5 min using a MiniSpin microcentrifuge (ThermoFisher Scientific). The supernatant was used as a template in the PCR assay immediately after extraction.

### 2.4. Detection of Virulence Genes in E. coli Isolates

Oligonucleotide primers targeting the *uid*A gene were used to confirm the isolates in the polymerase chain reaction with the conditions as shown in Table 1. PCR assay was carried out in a 25 μL reaction volume containing a 24 µL of 2×master mix (containing 0.05 U/µL of *Taq* DNA polymerase, reaction buffer, 4 mM MgCl_2_, and 0.4 mM of each dNTP) and 1µL of template DNA. Oligonucleotide primers targeting the *fliC*H7 gene encoding for structural flagella antigen H7 in Enterohemorrhagic *E. coli*, *eagR* gene encoding for antiaggregative protein of Enteroaggregative *E. coli, ial* gene encoding for invasion-associated locus of Enteroinvasive *E. coli, eae* gene encoding for Enteropathogenic *E. coli* intimin, *lt* gene encoding for heat-labile toxins found in Enterotoxigenic *E. coli* and *papC* gene characterizing uropathogenic *E. coli* were used in the polymerase chain reaction following the conditions in Table 1. The positive control for *uidA* was *E. coli* ATCC 8739 (SABS No. ESC 20) purchased from the South African Bureau of Standards in Pretoria; positive control for the *eagR* gene is DSM 10974, DSM 10973 for *lt* gene, DSM 4618 for *papC*, DSM 8695 for *eae* gene and DSM 9025 for *ial* gene and where all purchased from Leibniz-Institut DSMZ—Deutsche Sammlung von Mikroorganismen und Zellkulturen, Braunschweig, Germany. However the positive control for *fliCh7* gene (EHEC) was not available at the time of purchase. 

**Table 1:** Primer sequences and expected size of PCR amplified gene targets of the pathogenic strains of *Escherichia coli*.

Target Strain	Target Gene	Primer Sequence (5′–3′)	Conditions	Amplicon Size (bp)	References
*E. coli*	*UidA*	AAAACGGCAAGAAAAAGCAG ACGCGTGGTTAACAGTCTTGCG	An initial 2 min of denaturation at 94 °C followed by 25 cycles of 94 °C for 1 min, 58 °C for 1 min and 72 °C for 2 min. Amplified products were held at 4 °C after amplification.	147	[26]
EHEC	*flicH7*	TACCATCGCAAAAGCAAC TCC GTCGGCAACGTTAGTGATACC	Initial denaturation at 95 °C for 15 min followed by 35 cycles of heat denaturation at 94 °C for 45 sec, primer annealing at 55 °C for 45 sec and DNA extension at 68 °C for 2 min. After the last cycle the samples were kept at 72 °C for 5 min to complete synthesis of all strands.	230	[27]
EIEC	*Ial*	CTGGATGGTATGGTGAGG GGAGGCCAATTATTTCC	1 cycle for 2 min at 50 °C, 1 cycle for 5 min at 95 °C, 40 cycles for 45 sec at 95 °C, 45 sec at 55 °C and 45 sec at 72 °C and a final extension step for 10 min at 72 °C to complete synthesis of all strands.	700	[28]
EAEC	*EagR*	AGACTCTGGCGAAAGACTGTATC ATGGCTGTCTGTAATAGATGAGAAC	Initial denaturation at 95 °C for 15 min followed by 35 cycles of heat denaturation at 94 °C for 45 sec, primer annealing at 55 °C for 45 sec and DNA extension at 68 °C for 2 min. A final elongation step at 72 °C for 5 min.	194	[29]
EPEC	*EaeA*	TCAATGCAGTTCCGTTATCAGT GTAAAGTCCGTTACCCCAACC TG	An initial denaturation step at 94 °C for 5 min, followed by 36 cycles of 94 °C for 35 sec, annealing at 62 °C for 30 sec and elongation at 72 °C for 1 min. A final elongation step at 72 °C for 5 min.	482	[28]
ETEC	*Lt*	GCACACGGA GCTCCTCAGTC TCCTTCATCCTTTCAATGGCTT	An initial denaturation step at 94 °C for 5 min, followed by 36 cycles of 94 °C for 35 sec, annealing at 62 °C for 30 sec and elongation at 72 °C for 1 min. A final elongation step at 72 °C for 5 min.	218	[28]
UPEC	*PapC*	GACGGCTGTACTGCAGGGTGTGGCG ATATCCTTTCTGCAGGGATGCAATA	An initial denaturation step at 94 °C for 5 min, followed by 36 cycles of 94 °C for 35 sec, annealing at 62 °C for 30 sec and elongation at 72 °C for 1 min. A final elongation step at 72 °C for 5 min.	328	[30]

### 2.5. Gel Electrophoresis

The PCR products (10 μL aliquots) were resolved in 1.8% agarose gel containing 5 μL ethidium bromide in 1× TAE buffer (40 mM Tris-HCl, 20 mM Na-acetate, 1 mM EDTA, pH 8) [27,31] before being visualized and photographed under the Alliance 4.7 XD-79 System (Uvitec, Cambridge, UK). A 100-bp DNA ladder was included on each gel as a molecular size standard. The electrophoresis was carried out at 100 V for 1 h.

## 3. Results and Discussion

### 3.1. Isolation and Identification

#### 3.1.1. Biochemical Confirmation 

There were 100 (50%) Gram negative rods, catalase positive and oxidase negative in dairy farm A samples and 88 (44%) in dairy farm B. In dairy farm A, 72 (72%) were identified as *E. coli* using API 20E while dairy farm B, showed 68 (77%) *E. coli* (Table 2). Species identity by API ranged from 76.8% to 99.9% in dairy farm B and 76.8% to 100% in dairy farm A. 

**Table 2:** Table showing how organisms were identified from biochemical tests to molecular conformation.

Farms	Number of Samples		G (-ve) Rods	Cat (pos)	Ox (neg)	API 20E	*uidA* Gene
	Clinical Mastitis	Sub-Clinical Mastitis					
A	100	100	100	100	100	72	50
B	100	100	88	88	88	68	37

Legends: G (-ve) = Gram Negative; Cat (pos) = Catalase positive; Ox (neg) = Oxidase negative. All Gram negative rods were obtained from cows showing Clinical Mastitis.

#### 3.1.2. Molecular Characterization of *E. coli*

**Figure 1:**
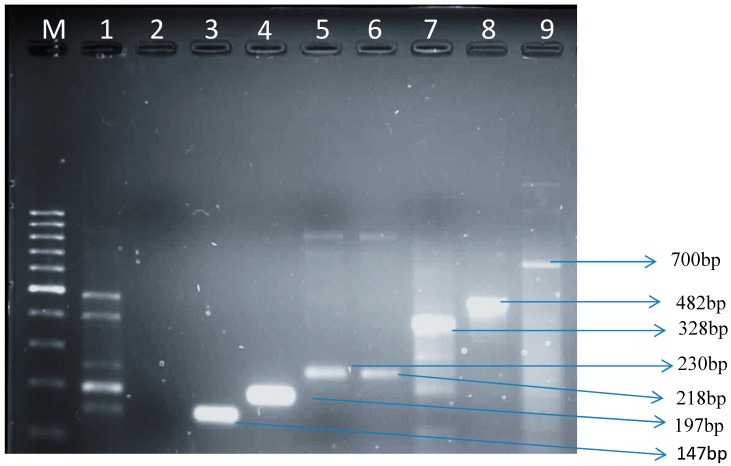
A representative gel showing all the genes amplified in this study.

Isolates that were Gram negative, oxidase negative, and catalase positive were screened for *E. coli* *uid*A gene using PCR assays. Dairy farm B showed 50 (57.5%) *uid*A gene amplification while dairy farm A showed 37 (42.5%) amplification (Table 2; Figure 1). Amplification of several genes representing *E. coli* pathotypes is shown in Figure 1. The *E. coli* isolates that were positive by PCR for *fliC*H7, *eae*, *lt, papC, ial,* and *eagR* genes are summarized in Table 3. Dairy farm A had the highest amount of virulence genes compared to dairy farm B except for *lt* gene, usually found in ETEC which was higher (13.5%) in farm B. The target gene of *E. coli* O157:H7 (*fliC*H7) was noticed in the isolates obtained from both dairy farms in high amounts in comparison to other virulence genes. Farm A is situated next to a wastewater treatment plant and the discharged final effluent is used to clean the clusters, milking machines, milking utensils and the floor of the milking parlour this could explain the high occurrence of pathogenic *E. coli* in this farm. Also the milking parlour is cleaned once a day in this farm, whereas in farm B it is cleaned twice a day after every milking activity. Cattle faeces were observed around the farm floor, milking and feeding equipments. Poor hygiene of feeding equipment and contaminated feed plays an important role in milk contamination.

**Table 3:** Occurrence of pathogenic *E. coli* isolates from two dairy farms as indicated by presence of the target gene marker.

Location	Amplified Genes						
	*uidA*	*fliCH7*	*eae*	*lt*	*papC*	*Ial*	*eagR*
Dairy farm A	50 (57.5%)	27 (54%)	9 (18%)	2 (4%)	3 (6%)	4 (8%)	5 (10%)
Dairy farm B	37 (42.5%)	16 (43.2%)	9 (24.3%)	5 (13.5%)	1 (2.7%)	2 (5.4%)	4 (10.8%)
Total	87 (100%)	43 (49%)	18 (21%)	7 (8%)	4 (4.6%)	6 (7%)	9 (10.3%)

M = A 100bp molecular marker; 1 = positive controls of 6 of the amplified genes (band 1—*E. coli* ATCC 8739; band 2—DSM 10974 (EAEC); band 3—DSM 10973 (ETEC); band 4—DSM 4618 (UPEC); band 5—DSM 8695 (EPEC);band 6—DSM 9025 (EIEC)); 2 = negative control (PCR assayed nuclease free water); 3 = *uid* gene (*E. coli*); 4 = *eagg* (EAEC) gene; 5 = *fliCh7* (EHEC) gene, 6 = *lt* (ETEC) gene; 7 = *papC* (UPEC); 8 = *eae* (EPEC) gene; 9 = *ial* (EIEC) gene.

Mastitis in lactating dairy cows is commonly caused by *E. coli* strains. The severity and course of mastitis differs enormously depending on the individual cow’s response. However, the strain virulence may also play a role. The ability of *E. coli* to pick up DNA from the environment and therefore virulence genes are well demonstrated [32]. *E. coli* has been regarded as an indicator of fecal contamination [33], however, in the milk industry; it is regarded as poor hygiene indicator and insufficient sanitary practices during milking [34]. Raw unpasteurized milk can be an important source of food-borne pathogens because it harbors a variety of microorganisms while bulk tank raw milk may be contaminated by udder excretion of infected animals [35]. Unpasteurized milk is used to produce several types of cheese [35,36]. Consumption of unpasteurized and pasteurized milk has been blamed for disease in humans [37].

#### 3.1.3. Virulence Genes from *E. coli* Isolates in Raw Cow’s Milk

Different virulence genes were isolated from *E. coli* in diarrheic stools and water [38]. We based our selection of virulence genes for this study on their association with *E. coli* strains causing extra-intestinal and intestinal infections. There are no biochemical difference between bovine mastitis *E. coli* strains and faecal strains. Approximately 30.3% of the isolates in our study did not show amplification of examined genes. This might be indication that *E. coli* strains associated with mammary gland infections may use different mechanisms to cause diseases [39]. In another study, *E. coli* isolates (10.7%) from bovine mastitis were found to have a single virulence gene [40]. Several virulence factors have been identified in bovine mastitis associated *E. coli* isolates [41] but many bovine mastitis isolates lack any of the virulence factors [42]. 

In our study all of the isolates were *stx*1 and *stx* 2 negative. This result is similar to the results of [43,44] where *stx*1 and 2 were not detected from bovine mastitis. This might not be a true reflection of the results since sub-types of *stx* 1 and *stx* 2 were not tested. The *fliC*H7 gene is one of the genes used as a marker to identify *E. coli* O157:H7 [45] has been amplified from 27 (54%) samples from farm A and 16 (43.2%) from dairy farm B. This gene has also been isolated in *E. coli* from water by Okoh and Osode [46]. Although *fliCH7* gene’s associated with mastitis has not been isolated from cows with mastitis in South Africa, its association with severe clinical symptoms, including hemolytic colitis, hemolytic-uremic syndrome (HUS) and diarrhea have been well established [47,48]. Yogurt made on a farm from pasteurized milk was implicated in people affected by *E. coli* O157:H7, causing HUS cases among children [49]. Therefore, its presence in raw milk is a cause for concern.

The *lt* gene which encodes for heat-labile toxin, found in Enterotoxigenic *E. coli* [50] was amplified from 2 (4 %) isolates in dairy farm B and 5 (13.5%) dairy farm A. These results are different from what Frank *et al.* [51] reported (3.2%) of ETEC strains in milk and milk products. As far as we know heat-labile toxin has never been shown to be associated with mastitis. However, the organism that produces heat-labile toxin, Enterotoxigenic *E. coli* (ETEC), causes infantile and travelers’ in humans from developed to underdeveloped countries [52]. Contaminated food including milk and water serves as a route of infection for ETEC [53]. The disease manifests as a severe cholera-like syndrome [53,54]. This gene has also been detected *E. coli* from water [38].

The *ial* gene (a marker for the virulence plasmid pInv) which is found in Enteroinvasive *E. coli* was also amplified from 4 (8%) isolates in dairy farm A and 2 (5.4%) isolates from dairy farm B. Contaminated food and water have been implicated in the transmission of EIEC [55,56] even though person-to-person cases of EIEC have been noted [57]. When this organism is ingested, invasion and adhesion of the epithelial cells in the intestine mediated by the *ial* gene occurs. The characteristics of the illness are the appearance of blood and mucus in a condition called colitis of infected individuals [58]. Diarrhea, abdominal cramps, fever, a generalized malaise, vomiting, chills, are some of the symptoms. Outbreaks have been associated with hamburger meat and unpasteurized milk [59]. This is the first report where EIEC has been isolated from raw milk in the Eastern Cape Province of South Africa. Enteroaggregative *E. coli* was isolated and *eagR* amplified from 5 (10%) isolates in dairy farm A and 4 (10.8%) isolates from dairy farm B. Human clinical, animal, and environmental samples are some of the samples where this organism has been increasingly isolated and characterized around the world [60,61]. However, frequencies of Enteroaggregative *Escherichia coli* among cattle in the Eastern Cape Province, South Africa, are not known. 

The virulence factor, *eae*, is associated with EPEC strains, defined as *Stx*-negative *E. coli* strains able to cause “attaching and effacing” (A/E) lesions, and also characterized by bacterial adherence to the intestinal epithelium intimately [62]. The *eaeA* and *bfp* genes have been used for identification of EPEC [63]. In our study, the ratio of *eae* was 9 (24.3%) in dairy farm B and 9 (18%) isolates in dairy farm A. However, in our study, we did not find any evidence of *bfp* in all the isolates tested. Our results inform us that the isolated organisms were atypical EPEC as they lacked the gene *bfp*. The presence of this pathogen in milk proved to be variable in different regions. In Brazil, Aleixo and Aver [64] reported EPEC in 25% milk samples while 26 (27.7%) from bulk milk were EPEC in Trinidad [65]. These values were higher than the 3.09% reported by Holko *et al.* [66] from Slovakia and that of Altalhi and Hussen [41], Saudi Arabia. EPEC strains are the oldest recognized category of diarrheagenic *E. coli* [67] and are well known prominent cause of diarrhea, particularly in children in less developed countries [68].

Uropathogenic *E. coli* (UPEC) which contain *papC* characterized as one of the pathogenic gene associated with the adhesion of UPEC to the upper urinary tract in humans [2] was amplified in three (6%) isolates from dairy farm A and one (2.7%) isolate in dairy farm B. The *papC* gene was reported to be associated with persistent mastitis by Dogan *et al.* [69]. Ingestion of milk contaminated with UPEC it can lead to cystitis in the bladder and acute pyelonephritis in the kidneys [3,70].

Act, No. 54 of 1972 in South Africa governs the safety of milk, and sets standards to which milk and dairy products, foodstuffs, cosmetics and disinfection must conform to. According to this Act milk may not contain pathogenic organisms, extraneous matter, any inflammatory product or any substance which may render it unfit for consumption. Bacteriologically it may not contain more than 20 coliforms or any *E. coli* per mL. This study indicates that raw milk samples from both dairy farm A and dairy farm B were not complying with the act and could be a course for public health concern as some pathogenic genes known to be associated with diarheagenic *E. coli* were detected. 

## 4. Conclusions

The results obtained in this study shows that raw cow’s milk available to consumers in the Eastern Cape, South Africa was contaminated with the pathogen *E. coli*. Regular washing of milker’s hands and animal udders together with sterilization of utensils and dairy equipment, is highly recommended as strict preventive measures. This might help in suppression of mastitis disease from the herd. Teaching and training programs for those working at the dairies can possibly improve the situation.
